# Ethyl 4-[(*E*)-(2-hy­droxy­benzyl­idene)amino]­piperidine-1-carboxyl­ate

**DOI:** 10.1107/S1600536811049750

**Published:** 2011-11-25

**Authors:** Rui-Qin Fang, Zhi-Li Shan, Xing Guo

**Affiliations:** a School of Life Science and Technology, University of Electronic Science and Technology of China, Chengdu 610054, People’s Republic of China; b State Key Laboratory of Pharmaceutical Biotechnology, Nanjing University, Nanjing, 210093, People’s Republic of China

## Abstract

In the title compound, C_15_H_20_N_2_O_3_, the piperidine ring adopts a chair conformation, although the amide N atom is almost planar (bond angle sum = 359.7°). The mol­ecule adopts an *E* conformation about the C=N bond, which allows for the formation of an intra­molecular O—H⋯N hydrogen bond. In the crystal, mol­ecules are linked by C—H⋯O inter­ations, resulting in *C*(6) chains propagating in [010].

## Related literature

For a related structure, see: Tas *et al.* (2007 ▶). For standard bond lengths, see: Allen *et al.* 1987) ▶.
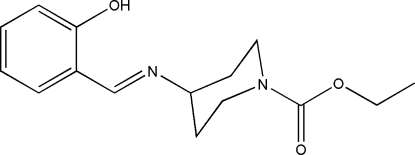


## Experimental

### 

#### Crystal data

C_15_H_20_N_2_O_3_
*M* *_r_* = 276.33
Monoclinic, 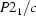

*a* = 15.732 (3) Å
*b* = 9.1890 (18) Å
*c* = 10.414 (2) Å
β = 97.24 (3)°
*V* = 1493.5 (5) Å^3^
*Z* = 4
Mo *K*α radiation
μ = 0.09 mm^−1^
*T* = 293 K
0.28 × 0.23 × 0.22 mm

#### Data collection

Enraf–Nonius CAD-4 diffractometer
Absorption correction: ψ scan (North *et al.*, 1968) ▶ *T*_min_ = 0.976, *T*_max_ = 0.981
3098 measured reflections
2922 independent reflections
1750 reflections with *I* > 2σ(*I*)
*R*_int_ = 0.032
3 standard reflections every 200 reflections  intensity decay: 1%

#### Refinement

*R*[*F* ^2^ > 2σ(*F* ^2^)] = 0.065
*wR*(*F* ^2^) = 0.174
*S* = 1.09
2922 reflections
183 parameters
H-atom parameters constrained
Δρ_max_ = 0.21 e Å^−3^
Δρ_min_ = −0.16 e Å^−3^

### 

Data collection: *CAD-4 Software* (Enraf–Nonius, 1989 ▶); cell refinement: *CAD-4 Software*; data reduction: *XCAD4* (Harms & Wocadlo, 1995 ▶); program(s) used to solve structure: *SHELXS97* (Sheldrick, 2008 ▶); program(s) used to refine structure: *SHELXL97* (Sheldrick, 2008 ▶); molecular graphics: *SHELXTL* (Sheldrick, 2008 ▶); software used to prepare material for publication: *SHELXL97*.

## Supplementary Material

Crystal structure: contains datablock(s) global, I. DOI: 10.1107/S1600536811049750/hb6521sup1.cif

Structure factors: contains datablock(s) I. DOI: 10.1107/S1600536811049750/hb6521Isup2.hkl

Supplementary material file. DOI: 10.1107/S1600536811049750/hb6521Isup3.cdx

Supplementary material file. DOI: 10.1107/S1600536811049750/hb6521Isup4.cml

Additional supplementary materials:  crystallographic information; 3D view; checkCIF report

## Figures and Tables

**Table 1:** Hydrogen-bond geometry (Å, °)

*D*—H⋯*A*	*D*—H	H⋯*A*	*D*⋯*A*	*D*—H⋯*A*
O1—H1⋯N1	0.82	1.87	2.592 (3)	146
C15—H15*B*⋯O2^i^	0.96	2.56	3.475 (5)	160

Symmetry code: (i) 
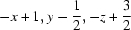
.

**Fig. 1.**
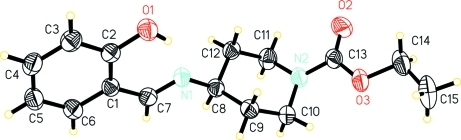
The structure of the title compound (I) showing 35% probability displacement ellipsoids.

**Fig. 2.**
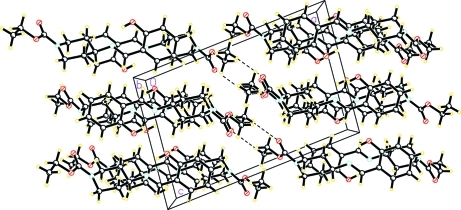
The crystal packing of (I), viewed along the b axis. Hydrogen bonds are shown as dashed lines.

## supplementary crystallographic information

### Comment

The cystal structure of the Schiff base *N*-(1-ethoxycarbonyl)piperidine-4-yl)-3,5-di-t-butylsalicylaldimine, derived from ethyl 4-aminopiperidine-1-carboxylate and 3,5-di-*tert*-butylsalicylaldehyde, has been reported before (Tas *et al.*, 2007). There are two *tert* butyl subsitituents on the 3- and 5- positons, as compared with the title compound. The molecular structure of title compound (I), Fig. 1, possesses an E configuration about C7=N1 double bond, and the bond length 1.268 (3) Å is in the normal range. (Allen *et al.* 1987). The C13=O2 double bond 1.208 (4) Å. The C2—O1, N2—C13 and C13—O3 single bond lengths are 1.352 (3), 1.343 (4) and 1.347 (4) Å, respectively. All these bond lengths is comparable to that observed in the reference compound. (Tas *et al.*, 2007) The torsion angle of C9—C8—N1—C7 and C12—C8—N1—C7 is -108.8 (3) ° and 131.3 (3) °. The Rms deviation of phenyl ring is 0.0072 Å, and the Rms of six-member piperidine ring of chair conformation is 0.2282 Å. The The dihedral angle between phenyl plane and piperidine ring in title compound is 77.58 (10) °. There is an intramolecular hydrogen bond, O1—H1···N1, together with one kind of intermolecular hydrogen bond C15—H15B···O2 in the crystal structure of title compound. All these hydrogen bonds the molecule to form an extended network along *b* axis, Fig. 2.

### Experimental

The title compound was prepared by stirring a mixture of salicylaldehyde (122 mg, 1 mmol) and ethyl 4-aminopiperidine-1-carboxylate (172 mg, 1 mmol) in methanol (15 ml) for 3 h at room temperature. After keeping the solution in air for 4 d, yellow block-shaped crystals of (I) were formed. The crystals were isolated, washed three times with methanol and dried in a vacuum desiccator containing anhydrous CaCl_2_.

### Refinement

All the H atoms, were placed in idealized positions (C—H = 0.93- 0.96 Å, O—H = 0.82 Å) and refined as riding with *U*_iso_(H) = 1.2*U*_eq_(C) and *U*_iso_(H) = 1.5*U*_eq_(O).

### Crystal data

**Table:**

C_15_H_20_N_2_O_3_	*F*(000) = 592
*M**_r_* = 276.33	*D*_x_ = 1.229 Mg m^−^^3^
Monoclinic, *P*2_1_/*c*	Mo *K*α radiation, λ = 0.71073 Å
Hall symbol: -P 2ybc	Cell parameters from 1428 reflections
*a* = 15.732 (3) Å	θ = 2.5–24.2°
*b* = 9.1890 (18) Å	µ = 0.09 mm^−^^1^
*c* = 10.414 (2) Å	*T* = 293 K
β = 97.24 (3)°	Block, yellow
*V* = 1493.5 (5) Å^3^	0.28 × 0.23 × 0.22 mm
*Z* = 4	

### Data collection

**Table:**

Enraf–Nonius CAD-4 diffractometer	1750 reflections with *I* > 2σ(*I*)
Radiation source: fine-focus sealed tube	*R*_int_ = 0.032
graphite	θ_max_ = 26.0°, θ_min_ = 1.3°
ω/2θ scan	*h* = −19→19
Absorption correction: ψ scan (North *et al.*, 1968)	*k* = −11→0
*T*_min_ = 0.976, *T*_max_ = 0.981	*l* = 0→12
3098 measured reflections	3 standard reflections every 200 reflections
2922 independent reflections	intensity decay: 1%

### Refinement

**Table:**

Refinement on *F*^2^	Secondary atom site location: difference Fourier map
Least-squares matrix: full	Hydrogen site location: inferred from neighbouring sites
*R*[*F*^2^ > 2σ(*F*^2^)] = 0.065	H-atom parameters constrained
*wR*(*F*^2^) = 0.174	*w* = 1/[σ^2^(*F*_o_^2^) + (0.0611*P*)^2^ + 0.6373*P*] where *P* = (*F*_o_^2^ + 2*F*_c_^2^)/3
*S* = 1.09	(Δ/σ)_max_ < 0.001
2922 reflections	Δρ_max_ = 0.21 e Å^−^^3^
183 parameters	Δρ_min_ = −0.16 e Å^−^^3^
0 restraints	Extinction correction: *SHELXL97* (Sheldrick, 2008), Fc^*^=kFc[1+0.001xFc^2^λ^3^/sin(2θ)]^-1/4^
Primary atom site location: structure-invariant direct methods	Extinction coefficient: 0.022 (3)

### Special details

**Table:**

Geometry. All e.s.d.'s (except the e.s.d. in the dihedral angle between two l.s. planes) are estimated using the full covariance matrix. The cell e.s.d.'s are taken into account individually in the estimation of e.s.d.'s in distances, angles and torsion angles; correlations between e.s.d.'s in cell parameters are only used when they are defined by crystal symmetry. An approximate (isotropic) treatment of cell e.s.d.'s is used for estimating e.s.d.'s involving l.s. planes.
Refinement. Refinement of *F*^2^ against ALL reflections. The weighted *R*-factor *wR* and goodness of fit *S* are based on *F*^2^, conventional *R*-factors *R* are based on *F*, with *F* set to zero for negative *F*^2^. The threshold expression of *F*^2^ > σ(*F*^2^) is used only for calculating *R*-factors(gt) *etc*. and is not relevant to the choice of reflections for refinement. *R*-factors based on *F*^2^ are statistically about twice as large as those based on *F*, and *R*- factors based on ALL data will be even larger.

### Fractional atomic coordinates and isotropic or equivalent isotropic displacement parameters (Å^2^)

**Table:**

	*x*	*y*	*z*	*U*_iso_*/*U*_eq_	
C1	−0.07284 (18)	0.1352 (3)	0.1715 (3)	0.0491 (7)	
C2	−0.11096 (19)	0.0759 (3)	0.2735 (3)	0.0503 (7)	
C3	−0.19939 (19)	0.0618 (3)	0.2628 (3)	0.0619 (8)	
H3	−0.2248	0.0232	0.3312	0.074*	
C4	−0.2494 (2)	0.1047 (4)	0.1517 (3)	0.0632 (9)	
H4	−0.3087	0.0962	0.1460	0.076*	
C5	−0.2130 (2)	0.1602 (4)	0.0488 (3)	0.0661 (9)	
H5	−0.2471	0.1874	−0.0268	0.079*	
C6	−0.1251 (2)	0.1749 (4)	0.0594 (3)	0.0624 (8)	
H6	−0.1003	0.2124	−0.0100	0.075*	
C7	0.01943 (18)	0.1592 (3)	0.1838 (3)	0.0515 (7)	
H7	0.0431	0.2014	0.1153	0.062*	
C8	0.16035 (17)	0.1521 (3)	0.2946 (3)	0.0526 (7)	
H8	0.1725	0.2147	0.2228	0.063*	
C9	0.2061 (2)	0.0066 (4)	0.2873 (3)	0.0645 (9)	
H9A	0.1913	−0.0348	0.2017	0.077*	
H9B	0.1865	−0.0601	0.3496	0.077*	
C10	0.3033 (2)	0.0227 (4)	0.3151 (3)	0.0677 (9)	
H10A	0.3300	−0.0725	0.3170	0.081*	
H10B	0.3242	0.0792	0.2469	0.081*	
C11	0.2866 (2)	0.2392 (4)	0.4458 (3)	0.0680 (9)	
H11A	0.3062	0.3029	0.3813	0.082*	
H11B	0.3038	0.2814	0.5305	0.082*	
C12	0.18953 (18)	0.2265 (3)	0.4225 (3)	0.0557 (8)	
H12A	0.1697	0.1713	0.4923	0.067*	
H12B	0.1644	0.3229	0.4224	0.067*	
C13	0.37125 (18)	0.0361 (4)	0.5437 (3)	0.0584 (8)	
C14	0.4508 (2)	−0.1707 (5)	0.6252 (4)	0.0828 (11)	
H14A	0.4184	−0.1817	0.6980	0.099*	
H14B	0.5018	−0.1140	0.6531	0.099*	
C15	0.4747 (3)	−0.3153 (5)	0.5780 (5)	0.1084 (15)	
H15A	0.4240	−0.3724	0.5553	0.163*	
H15B	0.5117	−0.3639	0.6449	0.163*	
H15C	0.5040	−0.3031	0.5032	0.163*	
N1	0.06833 (14)	0.1243 (3)	0.2853 (2)	0.0528 (6)	
N2	0.32588 (16)	0.0948 (3)	0.4385 (2)	0.0626 (7)	
O1	−0.06447 (13)	0.0300 (2)	0.38413 (19)	0.0671 (6)	
H1	−0.0133	0.0389	0.3780	0.101*	
O2	0.38554 (15)	0.0953 (3)	0.6478 (2)	0.0837 (8)	
O3	0.39911 (13)	−0.0984 (3)	0.5189 (2)	0.0702 (7)	

### Atomic displacement parameters (Å^2^)

**Table:**

	*U*^11^	*U*^22^	*U*^33^	*U*^12^	*U*^13^	*U*^23^
C1	0.0515 (17)	0.0451 (16)	0.0500 (17)	0.0037 (13)	0.0037 (13)	0.0011 (13)
C2	0.0551 (18)	0.0422 (16)	0.0534 (17)	0.0012 (13)	0.0065 (14)	−0.0017 (14)
C3	0.0563 (19)	0.061 (2)	0.070 (2)	−0.0036 (15)	0.0119 (16)	−0.0025 (17)
C4	0.0478 (17)	0.064 (2)	0.078 (2)	0.0019 (15)	0.0065 (17)	−0.0118 (18)
C5	0.059 (2)	0.069 (2)	0.066 (2)	0.0056 (17)	−0.0080 (17)	−0.0065 (17)
C6	0.063 (2)	0.070 (2)	0.0534 (18)	0.0038 (16)	0.0041 (15)	0.0062 (16)
C7	0.0537 (17)	0.0496 (17)	0.0518 (17)	−0.0005 (14)	0.0089 (14)	0.0064 (14)
C8	0.0487 (16)	0.0589 (18)	0.0506 (17)	0.0013 (14)	0.0076 (13)	0.0110 (14)
C9	0.063 (2)	0.071 (2)	0.0567 (18)	0.0102 (16)	−0.0036 (15)	−0.0106 (16)
C10	0.065 (2)	0.079 (2)	0.0577 (19)	0.0233 (18)	0.0004 (16)	0.0001 (17)
C11	0.059 (2)	0.062 (2)	0.080 (2)	−0.0032 (16)	−0.0050 (17)	−0.0067 (18)
C12	0.0566 (18)	0.0505 (17)	0.0603 (18)	0.0047 (14)	0.0087 (14)	0.0013 (15)
C13	0.0370 (15)	0.077 (2)	0.060 (2)	−0.0037 (15)	0.0008 (14)	0.0034 (18)
C14	0.060 (2)	0.105 (3)	0.079 (2)	0.005 (2)	−0.0110 (18)	0.025 (2)
C15	0.080 (3)	0.099 (3)	0.135 (4)	0.015 (2)	−0.029 (3)	0.034 (3)
N1	0.0464 (14)	0.0562 (15)	0.0551 (15)	0.0019 (11)	0.0036 (11)	0.0084 (12)
N2	0.0598 (16)	0.0654 (17)	0.0588 (16)	0.0088 (13)	−0.0075 (12)	−0.0021 (14)
O1	0.0574 (13)	0.0852 (16)	0.0590 (13)	−0.0027 (11)	0.0085 (10)	0.0187 (12)
O2	0.0713 (16)	0.113 (2)	0.0625 (15)	−0.0005 (14)	−0.0089 (12)	−0.0106 (15)
O3	0.0568 (13)	0.0798 (16)	0.0697 (14)	0.0101 (12)	−0.0085 (11)	0.0118 (12)

### Geometric parameters (Å, °)

**Table:**

C1—C6	1.389 (4)	C10—N2	1.450 (4)
C1—C2	1.394 (4)	C10—H10A	0.9700
C1—C7	1.458 (4)	C10—H10B	0.9700
C2—O1	1.352 (3)	C11—N2	1.470 (4)
C2—C3	1.388 (4)	C11—C12	1.520 (4)
C3—C4	1.373 (4)	C11—H11A	0.9700
C3—H3	0.9300	C11—H11B	0.9700
C4—C5	1.375 (4)	C12—H12A	0.9700
C4—H4	0.9300	C12—H12B	0.9700
C5—C6	1.380 (4)	C13—O2	1.208 (4)
C5—H5	0.9300	C13—N2	1.343 (4)
C6—H6	0.9300	C13—O3	1.347 (4)
C7—N1	1.268 (3)	C14—O3	1.449 (4)
C7—H7	0.9300	C14—C15	1.482 (6)
C8—N1	1.461 (3)	C14—H14A	0.9700
C8—C12	1.516 (4)	C14—H14B	0.9700
C8—C9	1.525 (4)	C15—H15A	0.9600
C8—H8	0.9800	C15—H15B	0.9600
C9—C10	1.527 (4)	C15—H15C	0.9600
C9—H9A	0.9700	O1—H1	0.8200
C9—H9B	0.9700		
			
C6—C1—C2	118.5 (3)	N2—C10—H10B	109.7
C6—C1—C7	120.7 (3)	C9—C10—H10B	109.7
C2—C1—C7	120.8 (3)	H10A—C10—H10B	108.2
O1—C2—C3	117.9 (3)	N2—C11—C12	110.1 (3)
O1—C2—C1	122.2 (3)	N2—C11—H11A	109.6
C3—C2—C1	119.9 (3)	C12—C11—H11A	109.6
C4—C3—C2	120.2 (3)	N2—C11—H11B	109.6
C4—C3—H3	119.9	C12—C11—H11B	109.6
C2—C3—H3	119.9	H11A—C11—H11B	108.2
C3—C4—C5	120.8 (3)	C8—C12—C11	111.2 (2)
C3—C4—H4	119.6	C8—C12—H12A	109.4
C5—C4—H4	119.6	C11—C12—H12A	109.4
C4—C5—C6	119.1 (3)	C8—C12—H12B	109.4
C4—C5—H5	120.5	C11—C12—H12B	109.4
C6—C5—H5	120.5	H12A—C12—H12B	108.0
C5—C6—C1	121.5 (3)	O2—C13—N2	124.8 (3)
C5—C6—H6	119.3	O2—C13—O3	123.8 (3)
C1—C6—H6	119.3	N2—C13—O3	111.3 (3)
N1—C7—C1	121.8 (3)	O3—C14—C15	107.4 (3)
N1—C7—H7	119.1	O3—C14—H14A	110.2
C1—C7—H7	119.1	C15—C14—H14A	110.2
N1—C8—C12	108.9 (2)	O3—C14—H14B	110.2
N1—C8—C9	108.2 (2)	C15—C14—H14B	110.2
C12—C8—C9	110.3 (2)	H14A—C14—H14B	108.5
N1—C8—H8	109.8	C14—C15—H15A	109.5
C12—C8—H8	109.8	C14—C15—H15B	109.5
C9—C8—H8	109.8	H15A—C15—H15B	109.5
C8—C9—C10	111.9 (3)	C14—C15—H15C	109.5
C8—C9—H9A	109.2	H15A—C15—H15C	109.5
C10—C9—H9A	109.2	H15B—C15—H15C	109.5
C8—C9—H9B	109.2	C7—N1—C8	120.2 (2)
C10—C9—H9B	109.2	C13—N2—C10	125.9 (3)
H9A—C9—H9B	107.9	C13—N2—C11	120.3 (3)
N2—C10—C9	109.9 (3)	C10—N2—C11	113.6 (2)
N2—C10—H10A	109.7	C2—O1—H1	109.5
C9—C10—H10A	109.7	C13—O3—C14	116.0 (3)
			
C6—C1—C2—O1	177.8 (3)	C9—C8—C12—C11	53.5 (3)
C7—C1—C2—O1	−4.2 (4)	N2—C11—C12—C8	−55.7 (4)
C6—C1—C2—C3	−1.9 (4)	C1—C7—N1—C8	−179.1 (3)
C7—C1—C2—C3	176.2 (3)	C12—C8—N1—C7	131.3 (3)
O1—C2—C3—C4	−179.0 (3)	C9—C8—N1—C7	−108.8 (3)
C1—C2—C3—C4	0.6 (4)	O2—C13—N2—C10	−175.8 (3)
C2—C3—C4—C5	0.9 (5)	O3—C13—N2—C10	4.1 (4)
C3—C4—C5—C6	−1.2 (5)	O2—C13—N2—C11	−2.3 (5)
C4—C5—C6—C1	0.0 (5)	O3—C13—N2—C11	177.6 (3)
C2—C1—C6—C5	1.6 (5)	C9—C10—N2—C13	115.9 (3)
C7—C1—C6—C5	−176.5 (3)	C9—C10—N2—C11	−58.0 (4)
C6—C1—C7—N1	179.7 (3)	C12—C11—N2—C13	−115.3 (3)
C2—C1—C7—N1	1.8 (4)	C12—C11—N2—C10	59.0 (4)
N1—C8—C9—C10	−172.0 (2)	O2—C13—O3—C14	−1.6 (4)
C12—C8—C9—C10	−53.0 (3)	N2—C13—O3—C14	178.5 (3)
C8—C9—C10—N2	54.5 (4)	C15—C14—O3—C13	−179.8 (3)
N1—C8—C12—C11	172.1 (2)		

### Hydrogen-bond geometry (Å, °)

**Table:**

*D*—H···*A*	*D*—H	H···*A*	*D*···*A*	*D*—H···*A*
O1—H1···N1	0.82	1.87	2.592 (3)	146
C15—H15B···O2^i^	0.96	2.56	3.475 (5)	160

Symmetry codes: (i) −*x*+1, *y*−1/2, −*z*+3/2.
